# Validation of the Erlangen Test of Activities of Daily Living in Persons with Mild Dementia or Mild Cognitive Impairment (ETAM)

**DOI:** 10.1186/s12877-016-0271-9

**Published:** 2016-05-26

**Authors:** Katharina Luttenberger, Simone Reppermund, Anke Schmiedeberg-Sohn, Stephanie Book, Elmar Graessel

**Affiliations:** Center for Health Services Research in Medicine, Department of Psychiatry and Psychotherapy, Friedrich-Alexander-Universität Erlangen-Nürnberg, Schwabachanlage 6, Erlangen, 91054 Germany; Department of Developmental Disability Neuropsychiatry (3DN), School of Psychiatry, University of New South Wales/ UNSW Medicine, 34 Botany Street, UNSW, Sydney, NSW 2052 Australia

**Keywords:** Mild cognitive impairment, Dementia, Activities of daily living, Performance test, Validity

## Abstract

**Background:**

There are currently no valid, fast, and easy-to-administer performance tests that are designed to assess the capacities to perform activities of daily living in persons with mild dementia and mild cognitive impairment (MCI). However, such measures are urgently needed for determining individual support needs as well as the efficacy of interventions. The aim of the present study was therefore to validate the Erlangen Test of Activities of Daily Living in Persons with Mild Dementia and Mild Cognitive Impairment (ETAM), a performance test that is based on the International Classification of Functioning and Health (ICF), which assesses the relevant domains of living in older adults with MCI and mild dementia who live independently.

**Methods:**

The 10 ICF-based items on the research version of the ETAM were tested in a final sample of 81 persons with MCI or mild dementia. The items were selected for the final version in accordance with 6 criteria: 1) all domains must be represented and have equal weight, 2) all items must load on the same factor, 3) item difficulties and item discriminatory powers, 4) convergent validity (Bayer Activities of Daily Living Scale [B-ADL]) and discriminant validity (Mini Mental State Examination [MMSE], Geriatric Depression Scale 15 [GDS-15]), 5) inter-rater reliabilities of the individual items, 6) as little material as possible. Retest reliability was also examined. Cohen’s ds were calculated to determine the magnitudes of the differences in ETAM scores between participants diagnosed with different grades of severity of cognitive impairment.

**Results:**

The final version of the ETAM consists of 6 items that cover the five ICF domains communication, mobility, self-care, domestic life (assessed by two 3-point items), and major life areas (specifically, the economic life sub-category) and load on a single factor. The maximum achievable score is 30 points (6 points per domain). The average administration time was 35 min, 19 of which were needed for pure item performance. The internal consistency was α = .71. The three-week test-retest reliability was *r* = .78, and the inter-rater reliability was *r* = .97. The ETAM also provided satisfactory discrimination between healthy individuals and persons with MCI or mild dementia as well as between persons with mild and moderate dementia.

**Conclusions:**

The 6-item final version of the ETAM shows satisfactory psychometric characteristics and can be administered quickly. It is therefore suitable for use in both clinical practice and research.

**Electronic supplementary material:**

The online version of this article (doi:10.1186/s12877-016-0271-9) contains supplementary material, which is available to authorized users.

## Background

In addition to cognitive deficits and behavioural problems, a decline in the capacity to perform activities of daily living (ADL and instrumental ADL [IADL]) is a central marker for the presence of dementia. Intact capacities for performing activities of daily living are decisive for the autonomy of individuals with dementia and thus also for their quality of life [1–3]. According to the International Classification of Functioning (ICF) [4], in the domain of “Activity and Participation”, the five behavioural areas of communication, mobility, self-care, domestic life, and major life areas (specifically, the economic life sub-category) are particularly important for older adults [4–6].

There is ample scientific evidence that people with mild dementia already have limited abilities to perform complex activities of daily living. Pérès et al. [6] demonstrated in their prospective study that initial impairments in performing more demanding activities of daily living can be detected even as early as ten years before the first clinical diagnosis of dementia. It has in fact been shown that IADL might already be impaired in the early stages of cognitive decline, even before a diagnosis of dementia is warranted [7–10]. MCI can be regarded as a transitional state that falls between normal aging and dementia, with a high probability of progressing to dementia [11]. The likelihood of progressing to dementia is significantly higher for individuals with MCI whose IADL are impaired, and the interval before developing dementia is significantly shorter than in MCI in the absence of IADL impairment [12–14]. This seems to be the case even when cognitive function is controlled for [15].

Since people with MCI or mild dementia are particularly likely to still be living at home in their own households, an early and exact diagnosis of their capacities to engage in activities of daily living is necessary in order to be able to estimate risks and to implement suitable treatment quickly. However, despite the great importance of the ability to perform activities of daily living for personal autonomy in persons with MCI and mild dementia, until now, there have been very few instruments that can adequately measure ADL capacities. In his review [16], Gold reported on a number of observer rating scales such as the Alzheimer’s Disease Cooperative Study Scale for ADL in Mild Cognitive Impairment (ADCS-MCI-ADL) [17]. For mild dementia, there is the Bayer Activities of Daily Living Scale (B-ADL) [18]. However, it is important to note that the ADCS-MCI-ADL and the B-ADL are proxy ratings for determining the capacity to engage in activities of daily living for persons with mild to moderate dementia, and there is no validated performance test that can be used to assess MCI or mild dementia [19].

Although performance tests generally take more time to administer and require more resources, such tests provide standardised and thus more objective results [20]. Thereby, they offer a solution to the problem that the proxy ratings mentioned above are often subject to rater biases (e.g., raters who are relatives of individuals with MCI or mild dementia tend to under-estimate deficits in activities of daily living [21, 22]; also, the rater’s mood and subjective care burden can influence the assessment outcome [21, 23]). The few measures of activities of daily living in dementia that have been available to date have some marked disadvantages. Most of them take between 45 min (Functional Living Skills Assessment [FLSA] [24]) and 1.5 h (Direct Assessment of Functional Abilities [DAFA] [25]) to administer. These long administration times are often due to the complex test instructions and the elaborate preparations that are required. The validation samples are usually very small and selective, such as those used for the Structured Assessment of Independent Living Skills (SAILS) [26] (only 18 dementia patients). An exception is the Test of Everyday Functional Abilities (TEFA) [27] (15–20 min on average); however, its results are largely consistent with those of the MMSE [27, 28]. Thus, the TEFA cannot differentiate to a satisfactory degree between ADL and cognitive abilities (correlation greater than .9).

Another disadvantage of these tests is that they cover only a limited range of relevant domains of activities of daily living. In addition, the Direct Assessment of Functional Status (DAFS) [29] and DAFS-R [30] include a number of items that are culture-specific and therefore cannot be administered in many European countries as they pertain either to the American Health Services System (e.g., refilling a prescription) or to other specifics of North-American life (e.g., dialling the operator).

None of the existing performance tests have been validated for MCI. All tests have marked ceiling effects in the area of mild dementia and are therefore more suitable for use with patients with moderate to severe dementia.

The aim of the present project was therefore to develop a performance test for persons with MCI or mild dementia, the contents of which would be oriented towards the ICF and which would thus measure a broad spectrum of abilities that are relevant for the performance of activities of daily living. This test was designed to be a sensitive measure of incipient deficits in activities of daily living and also to be fast and easy to administer.

## Methods

### Preliminary work

In a pilot study, a multi-step process was carried out to develop items that would correspond to the ICF domains or chapters “mobility”, “communication”, “self-care”, “domestic life”, and “major life areas” (sub-category: “economic life”) from the ICF category “Activities and Participation”. The acceptance and item characteristics of the resulting items and the time required for administration were investigated in 30 persons. For a more detailed description see [31]. The results of this study provided some initial indications of positive psychometric characteristics and economy in terms of the amount of resources required and a short administration time. On the basis of these results, problematic items were re-developed or modified in accordance with the same criteria. The research version of the ETAM used in the present study consisted of the items listed in Table 1.

**Table 1:** Research version of the ETAM

ICF domain “Activities and Participation”	Item (maximum achievable total score)	Task areas
Chapter 3: Communication	Phone call (6 points)	Finding a telephone number in the phone book. Making a call with a mobile phone for older adults, listening to and reporting the text of a voicemail.
Chapter 4 Mobility	Traffic situations (6 points)	Understanding basic rules in road traffic situations on the basis of example situations (e.g., traffic lights)
	Train timetable (3 points)	Calculating the time before the train comes and the duration of the train ride
Chapter 5 Self-care	Medication indication (6 points)	Assigning a particular medication to an indication (pain killers, cough medicine, for stomach problems)
	Medication expiry (6 points)	Checking how long a medication can still be used (using the expiry date)
	Pill organiser (6 points)^a^	Placing medications in a pill organiser according to a predefined schedule, for 4 different times of day for a particular day
Chapter 6 Domestic life	Making tea (3 points)	Making a cup of tea with a kettle
	Alarm clock (3 points)	Reading and setting times
	Washing the dishes (6 points)	Washing and drying the teacups that have been used
Chapter 8 Major life areas – economic life	Finances (6 points)	Comparing offers, adding up sums of money, counting money

^a^This item was formerly called “medication box” in the pilot study [31]

### Design

To validate and select the final items for the ETAM, we conducted a cross-sectional study. A total of 81 subjects with MCI or mild dementia from 10 supported-living institutions and 4 day-care centres in Middle Franconia (Bavaria, Germany) were included in the final sample. The project was funded by the German Research Foundation (DFG, Funding Number LU 1861/1-1). If the criteria for inclusion were fulfilled (described below), the research version of the ETAM was administered, after which the subjects were requested to fill out the Geriatric Depression Scale (GDS-15) [32]. We randomly selected 3 institutions with a total of 18 cases (about 20 % of the final sample), and a second, independent tester was employed to test the inter-rater reliability. In addition, 45 participants from 6 institutions (about 50 % of the final sample) were tested again about 3 weeks after the first administration to determine the test-retest reliability. All tests, including the screening tests, were administered by independent external testers who had undergone training in the administration of the scales and tests that were employed.

At the same time, for each participant, the person who was best informed about the participant’s capacities for performing activities of daily living was requested to complete the Bayer-ADL. This was either a family caregiver or a member of the care staff at the residential institution who had already known the participant for several months.

The study procedure was examined and approved by the ethics committee of the medical faculty of the University of Erlangen-Nuremberg (Approval Number 233_13B).

### Criteria for inclusion and exclusion

Persons who were potentially suitable participants for the study were thoroughly informed about the study procedure by the staff at the institutions. Either the potential participants themselves or their legal guardians were asked for consent. Written consent was obtained from all participants in the study and their relatives and, when applicable, their guardians. The Mini-Mental-State-Examination (MMSE) [33] and the Montreal Cognitive Assessment (MoCA) [34] were administered to all participants who had given their consent. The criteria for inclusion in the final sample consisted of a score of 19 or higher on the MMSE and, at the same time, a score of less than 23 points on the MoCA [35]. Participants with MMSE scores between 19 and 23 (inclusive) were those with cognitive deficits with the degree of severity of mild dementia, while participants with an MMSE score of 24 or higher and a MoCA result of less than 23 were assigned to the group of subjects with MCI [35].

In order to obtain an initial indication of the sensitivity of the ETAM, subjects with no cognitive deficits (MoCA >22) (the cut-off for MCI vs. no cognitive impairment) or who met the criteria for moderate dementia (cut-off for mild dementia vs. moderate dementia) (MMSE between 10 and 18) were also included in the study.

**Fig. 1:**
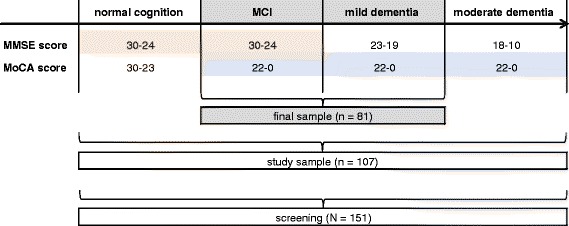
Description of the subsamples

Combining the MMSE and the MoCA enabled us to differentiate between our final sample (i.e., participants with either MCI or mild dementia) and the two other groups (i.e., participants with normal cognition and moderate dementia) (Fig. 1).

The criteria for exclusion were (1) a psychiatric diagnosis that could explain the cognitive deficits as attributable to a cause other than MCI or dementia (e.g., Korsakov’s syndrome), (2) paralysis of the upper limbs, or (3) strongly impaired hearing or vision.

### Measures

#### Tool under investigation

The research version of the ETAM consisted of 10 items (see Table 1) specifically developed for MCI/mild dementia [31]. The ETAM addresses the capacity to accomplish complex activities of daily living that cover the areas of living relevant to older adults living alone, i.e., communication, mobility, self-care, domestic life, and economic life (a major life area) from the ICF domain “Activities and Participation” [5]. The subject’s performance is judged on a four- or seven-point Likert scale (0–3 or 0–6 points). Each item is divided into 3 or 6 parts in such a way that participants receive 1 point for each part. The total score for the research version of the ETAM ranges from 0 to 51 points.

#### Control tools

The Mini-Mental State Examination (MMSE) [33] is the best-known and the most frequently used short screening tool for dementia [36]. It can be used to assess the progression and severity of cognitive impairment. It is a brief (5–10 min) 11-question measure that tests five areas of cognitive function: orientation, registration, attention and calculation, recall, and language. The total score ranges from 0 to 30 points, with higher values indicating a greater performance capacity. Scores ranging from 19 to 23 points are considered indicative of mild dementia; scores from 10 to 18 points, moderate dementia; and scores from 0 to 9 points, severe dementia. We used the German version by Kessler et al. [37].

The Montreal Cognitive Assessment (MoCA) [34] is a brief screening instrument for Mild Cognitive Impairment and has been shown in several studies to be more sensitive to detecting Mild Cognitive Impairment than the MMSE [34, 35, 38, 39]. Like the MMSE, MoCA scores range from 0 to 30 points. In contrast to the MMSE, the MoCA consists of more complex tasks, including executive function. For mixed samples of persons with and without cognitive complaints, a cut-off score of 26 was suggested in the original paper [34], whereas for more homogenous groups of persons with cognitive complaints, the specificity may increase if a lower cut-off is used [38, 40]. The validation study carried out by Freitas et al. [35] suggested that for MCI, a cut-off of 22 would be best with regard to both sensitivity and specificity and negative and positive predictive values. We used the German version available from http://www.mocatest.org.

The Bayer - Activities of Daily Living (Bayer-ADL) Scale [18] is an observer rating scale for determining the capacity for activities of daily living of persons with mild to moderate dementia. A person’s skills in activities of daily living are judged by that person’s main significant others. Basic capacities for performing activities of daily living are assessed on a 10-point scale for each item. The items include difficulties with personal hygiene and also complex activities such as organizing a household. A global value is calculated from the item values as a quotient of the total of the item values and the number of item responses. The greater the deficits in activities of daily living, the higher the overall score on the Bayer-ADL, which ranges from 1 to 10 points. For the purposes of this study, the German version by Erzigkeit et al. was used [41].

The Geriatric Depression Scale - 15 (GDS-15) [32] is a self-rating scale for measuring depressive symptoms in older adults. Both the long and short versions have good psychometric properties [32]. The GDS-15 can thus also be employed in patients with mild to moderate cognitive deficits [42]. The total score ranges from 0 to 15 points. Total scores over ten are indicative of a clinical manifestation of depression. The German version by Gauggel et al. was used in the current study [43].

### Statistical analyses

#### Decision-making criteria for selecting items

The following criteria were defined for selecting the items from the research version for the final version of the ETAM:

#### Criterion 1: all domains must be represented and have equal weight

Each ICF domain must be represented by at least one item, and all domains must be represented by the same number of points. Thus, the items that had the best scores in each domain were selected.

#### Criterion 2: factor analysis

All items must load on the same factor. There are numerous indications in the literature that ADL/IADL capacities in persons with dementia load mainly on a single common factor (a general ADL factor). Thus, in 2011, in their study on a “Capacity and Performance Scale”, which was based on the ICF, Almansa et al. [44] confirmed the unidimensionality of the scale “Activities and Participation” (one general factor and two additional “psychosocial” and “physical” factors). Findings by Erzigkeit et al. [45] and Voigt-Radloff et al. [46] from the validation of interviews with persons suffering from mild to moderate dementia also indicated a single general factor. We therefore assumed that such skills would be influenced by a general IADL/ADL factor and conducted an exploratory factor analysis with Kaiser normalisation. Items that deviated markedly from the main factor were excluded.

#### Criterion 3: item difficulties must fall in the range .2 ≤ p_i_ ≤ .8, and item discriminatory powers must be r_it_ ≥ .3

Item difficulties must fall in the range .2 ≤ p_i_ ≤ .8, and item discriminatory powers must be r_it_ > .3. The difficulty indices and discrimination powers were calculated at the item level. Because a 4- or 7-step response format (0–3 points or 0–6 points) was used for the ETAM items, the ratio of the sum of the subject’s points squared to the sum of the squared item maximum ($\frac{{\displaystyle \sum {x}^2}}{{\displaystyle \sum {x}_{\max}^2}}$) [47] was used as the difficulty index. Values of .2 ≤ p_i_ ≤ .8 were expected. Discrimination power was calculated as the corrected item-total correlation. According to Bortz and Döring [48], a discrimination power of .3 to .5 should be rated as moderate, whereas a discrimination power > .5 should be rated as high.

#### Criterion 4: items must have convergent and discriminant validity

Items must demonstrate convergent and discriminant validity. Items that showed correlations of not less than .2 with the Bayer ADL and were not more than moderately correlated with the MMSE (.5) were given preference. Item correlations with the GDS-15 were not to exceed .2. The correlations of the individual items with the above-mentioned tests were calculated with the Spearman rho formula.

#### Criterion 5: inter-rater reliabilities of the individual items must not be less than .8

The inter-rater reliabilities of the individual items must be not less than .8. A second, independent rater was employed for 20 % of the sample. The agreement was calculated in the form of correlations (Spearman’s rho).

#### Criterion 6: as little material as possible and quick administration

The fastest possible administration time and as little material as possible should be considered per item. When items were equally satisfactory according to the other criteria, the items that required less time to administer and/or less material were selected.

### Final version of the ETAM

The final version of the ETAM obtained using the above-mentioned criteria was tested to determine whether it was normally distributed (K-S test). We calculated the average amount of time it took to administer the items and the time to administer the test as a whole calculated. The psychometric properties of each item on the reduced version were calculated with the procedure described above.

The inter-item correlations (Spearman’s rho) were also calculated. For the total score for the final version of the ETAM, the correlations between the ETAM and the B-ADL, MMSE, and GDS-15 were also calculated. If there were no indications that the assumption of normally distributed values had been violated, Pearson correlations were computed.

Cronbach’s alpha was computed as a measure of internal consistency. For performance tests, α >.9 is considered to demonstrate excellent internal consistency, α >.8 good internal consistency, and α >.7 satisfactory internal consistency [49]. We also calculated the test-retest reliability after 3 weeks and the inter-rater reliability for the total score.

In order to assess the extent to which the ETAM could discriminate between different levels of severity of cognitive impairment, a one-way ANOVA with the total ETAM score as the dependent variable and the severity of cognitive impairment (unimpaired, MCI, mild or moderate dementia) as the independent variable was computed.

Cohen’s d [50] was used to examine the magnitude of the difference in ETAM scores between participants diagnosed with mild and moderate dementia and those with MCI and normal cognition. In addition, the area under the ROC curve was calculated to differentiate between MCI and healthy cognition.

## Results

### Sample

**Fig. 2:**
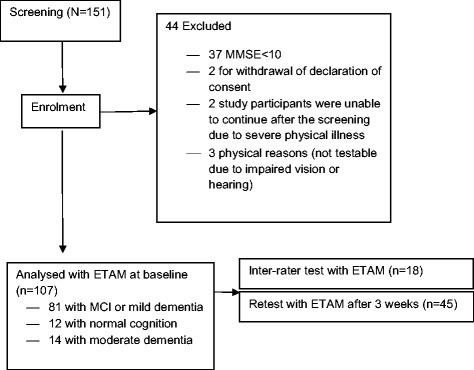
Flow chart showing how the sample was enrolled

A total of 151 persons were screened. Forty-four fulfilled at least one of the criteria for exclusion, in most cases severe dementia (Fig. 2). The study sample consisted of 107 participants, including 74 women (69 %) and 33 men (31 %). A total of 81 (76 %) participants met the study criteria for MCI or mild dementia (see Table 2), 12 were cognitively unimpaired (11 % of the study sample), and 14 showed a degree of cognitive impairment consistent with moderate dementia (13 % of the study sample). Most of the results were based on the final sample, consisting of 81 study participants with MCI or mild dementia; any deviations from this rule are mentioned explicitly.

**Table 2:** Sample characteristics

Characteristics	Total (*n* = 107)	MCI+MD (*n* = 81)
Age, mean (SD)	82.8 (8.0)	82.2 (8.1)
Women, no. (%)	74 (69.2)	54 (66.7)
Education, no. (%)^a^		
−8 years of schooling	45 (56.3)	31 (28.3)
−11 years of schooling	26 (32.5)	20 (24.7)
−13 or more years of schooling	9 (11.3)	8 (9.9)
Marital status, No. (%)^b^		
Married	21 (20.2)	13 (16.0)
Widowed	65 (60.7)	51 (63.0)
Divorced	7 (6.5)	5 (6.2)
Single	11 (10.3)	9 (11.1)
Care level, No. (%)^c^		
None	26 (24.3)	18 (22.2)
1	43 (40.2)	32 (39.5)
2	24 (36.7)	19 (23.5)
MMSE, mean (SD)	22.4 (4.4)	23.02 (3.0)
GDS-15, mean (SD)^d^	4.4 (3.4)	4.7 (3.4)
B-ADL, mean (SD)^e^	5.5 (2.4)	5.6 (2.3)

*MCI+MD* persons with mild cognitive impairment and persons with mild dementia, *MMSE* mini-mental status examination, *GDS-15* geriatric depression scale-15, *B-ADL* Bayer activities of daily living-scale

^a^Details on the education of 80 participants

^b^Family status of 104 study participants

^c^Details on the levels of care of 93 study participants

^d^GDS-15 details of 106 study participants

^e^B-ADL details of 105 study participants

### Research version

All items were tested according to the criteria defined above in order to reduce the number of items. Since Criterion 1 (All domains must be represented and have equal weight) was given the highest priority, we first report the results for Criteria 2–6 and then those for the individual domains.

#### Criterion 2: factor analysis

As shown in Table 3, the results of the factor analysis revealed a solution with two factors in which all ETAM items apart from the items “Medication indication” and “Washing the dishes” loaded on the same factor.

**Table 3:** Exploratory factor analysis of the research version (*n* = 81)

ICF Domain	Item	Component	
		1	2
Communication	Phone call	.64	.08
Mobility	Traffic situations	.68	−.08
	Bus timetable	.80	.06
Self-care	Medication indication	.10	.68
	Medication expiry	.68	.28
	Pill organiser	.58	.55
Domestic life	Making tea	.55	.18
	Alarm clock	.42	.51
	Washing the dishes	.07	.77
Major life areas - economic life	Finances	.52	.37

#### Criterion 3: item difficulties must fall in the range .2 ≤ p_i_ ≤ .8, and item discriminatory powers must be r_it_ ≥ .3

The item difficulties for the respective items ranged from .17 to .86. Apart from the items “Phone call” and “Medication indication”, all items were within the reference range of .2 to .8 (see Table 4). Apart from the items “Washing the dishes” (p_i_ = .26), the discriminatory powers of all items were r_it_ ≥.3 as required (see Table 4).

**Table 4:** Item characteristics of the research version of the ETAM (*n* = 81)

ICF domain	Item	Difficulty	Discriminatory power	Correlation with MMSE^a^	Correlation with GDS-15^b^	Correlation with B-ADL^c^	Inter-rater-correlation (*n* = 18)
Communication	Phone call	.17	.45	.33	.04	−.22	.95
Mobility	Traffic situations	.32	.41	.19	.05	−.41	.96
	Train timetable	.25	.59	.33	.07	−.31	1.00
Self-care	Medication indication	.86	.33	.23	.07	−.12	1.00
	Medication Expiry	.53	.60	.45	.11	−.47	.94
	Pill organiser	.40	.68	.38	.01	−.25	1.00
Domestic life	Making tea	.46	.44	.45	.13	−.14	.91
	Alarm clock	.42	.51	.28	−.13	−.28	.92
	Washing the dishes	.68	.26	.14	.08	−.14	.94
Major life areas-economic life	Finances	.52	.54	.34	.07	−.25	.98

^a^Mini-Mental Status Examination

^b^Geriatric Depression Scale

^c^B-ADL

#### Criterion 4: items must have convergent and discriminant validity

The correlations of the ETAM items with the B-ADL were between −.12 (“Medication indication”) and −.47 (“Medication expiry”). The maximum correlation with the MMSE on the item level was *r* = .45 (“Medication expiry” and “Making tea”). The maximum item correlation with the GDS-15 was |*r*| = .13 (“Alarm clock” and “Making tea”), thus also fulfilling the criteria. The correlations between the items on the research version of the ETAM and the MMSE, GDS-15, and B-ADL are also shown in Table 4.

#### Criterion 5: inter-rater reliabilities of the individual items must not be less than .8

All items achieved an inter-rater reliability of greater than .9 (see Table 4).

#### Criterion 6: as little material as possible and quick administration

The items “Washing the dishes” and “Making tea” required the most material. The items “Medication expiry” and “Train timetable” were also problematic since they had to be prepared anew for each test occasion (“Medication expiry”) or location (“Train timetable”). The items “Alarm clock”, “Medication indication”, and “Washing the dishes” required the shortest time (an average of approximately 2 min), followed by “Medication expiry” and “Train timetable” (2–3 min). “Making tea”, “Traffic situations”, “Phone call”, and “Finances” required an average of 3–4 min (these times refer to the time participants spent answering the item, not including preparation or verbal exchanges).

### Item reduction

#### Communication domain

Since it was not necessary to alter the “Phone call” item after the pilot study, there was no second item to provide a choice in this case. Apart from having a slightly elevated item difficulty (.17 instead of .20), the “Phone call” item met the criteria.

#### Mobility domain

The two items on Mobility, “Train timetable” and “Traffic situations”, differed only slightly in their discriminatory power and difficulty. However, the item “Train timetable” showed a stronger correlation with the MMSE and required more material because the timetable had to be compiled separately for each region. The “Train timetable” item was excluded.

#### Self-care domain

The item “Medication indication” was discarded on the basis of the results of the factor analysis, its low difficulty, and its weak correlation with the B-ADL. Most of the psychometric properties of the items “Medication expiry” and “Pill organiser” were similar. As the item “Pill organiser” was only weakly correlated with the MMSE and GDS-15, we decided to retain the self-care domain. This item can also be considered superior in terms of administration time, amount of material required, and its inter-rater correlation.

#### Domestic life domain

The item “Washing the dishes” showed poor factor analytic results, poor discriminatory power, and a low item difficulty; it also required a large amount of material to administer. It was therefore not included in the final version. The item “Alarm clock” showed satisfactory characteristics and was integrated into the final version on account of its high degree of practical relevance, short administration time, and the small amount of material it required. The item “Making tea” also showed satisfactory characteristics. Each of these items yielded only three points. They were therefore included in the final version because, in this way, the “Domestic Life” domain contributed a total of 6 possible points, like all the other domains.

#### Economic life (major life area)

The “Finances” item was also not modified after the pilot study. Its psychometric properties fulfilled the criteria defined above. This item could thereby be included on the final version.

### The final version

The final version of the ETAM consists of 6 items that represent the five relevant domains of the ICF (for the test evaluation sheet and the material, see Additional files 1 and 2). In order to sufficiently represent the broad range of activities covered by the Domestic life domain and due to the low degree of complexity of its two items “Making tea” and “Alarm clock”, these two items each contribute only 3 points to the overall score. For each of the other four items, a maximum of six points can be scored. Thus, each domain contributes a total of six points, adding up to a total possible score of 30 points across the five domains. The duration of administration is 19–35 min.

In the validation study, participants scored an average of 15.4 points with a standard deviation of 7.1. The median was 15.0 points. The distribution had a skewness of −.063 and a kurtosis of −.981. Only the minimum number of points (0) was not included in the distribution. Thus, at 1 to 30, the maximum range was almost completely covered. At the item level, the ranges of 0–3 and 0–6 were achieved for all items. The corresponding values at the item level and for the total score are shown in Table 5.

**Table 5:** Distribution values for ETAM scores and the items used on the final version

ICF domain and Items							
	Communication	Mobility	Self-care	Domestic life		Major life areas -economic life	
	Phone call	Traffic situations	Pill organiser	Making tea	Alarm clock	Finances	ETAM total score
Mean (Min; Max)	2.0 (0; 6)	3.0 (0; 6)	2.9 (0; 6)	1.9 (0; 3)	1.7 (0; 3)	4.0 (0; 6)	15.4 (1; 30)
Standard deviation	1.5	1.6	2.5	0.8	1.0	1.6	6.2
Skewness	1.05	−.18	.04	−.40	.11	−.68	−.08
Kurtosis	.56	−.58	−1.75	−.29	−1.16	−.14	−1.12
Discriminatory power of item	.42	.40	.64	.43	.43	.47	
Cronbach’s alpha if item deleted	.68	.68	.61	.69	.68	.65	

No significant deviation from a normal distribution for the distribution of the total ETAM score (*p* = .215) was found when computing the Kolmogorov-Smirnov test.

The mean overall administration time from the welcoming of the participants to their departure was 35 min, 19 of which were required for answering the items.

The 6 ETAM items were all positively and usually moderately correlated with each other (from *r* = .19 for “Traffic situations” and “Alarm clock” to *r* = .49 for “Pill organiser” and “Finances”). The correlations are shown in Table 6.

**Table 6:** Item correlations (Spearman) for the final version

ICF domain and Items						
	Communication	Mobility	Self-care	Domestic life		Major life areas -economic life
	Phone call	Traffic situations	Pill organiser	Making tea	Alarm clock	Finances
Phone call		.22	.38	.21	.29	.35
Traffic situations			.35	.22	.19	.37
Pill organiser				.39	.46	.49
Making tea					.34	.27
Alarm clock						.26

The Pearson correlations for the total ETAM score with the B-ADL (*r* = −.41) and the MMSE (*r* = .46) were both moderately strong.

The Pearson correlation between the total ETAM score and the GDS-15 was .05; thus, the two tests are not correlated.

Cronbach’s alpha was .71. The 3-week test-retest reliability of the ETAM was *r* = .78. The inter-rater reliability was .97.

If we grouped the study participants according to severity on the basis of their MMSE and MoCA values (no cognitive impairment, MCI, mild dementia, moderate dementia), significant differences between these groups were shown on the ETAM (ANOVA *p* < .001), i.e., the two capacities decreased in parallel. The arithmetic mean of the total ETAM score for the cognitively unimpaired older adults (*n* = 12) was 22.3 points, with a 95 % confidence interval (95 % CI) of 19.9–24.8. Persons with MCI (*n* = 44) scored on average 17.8 points (95 % CI 12.1–19.5). Participants with mild dementia (*n* = 37) achieved a mean of 12.7 points (95 % CI 10.6–14.8), while the mean for individuals with moderate dementia (*n* = 14) was 7.2 points (95 % CI 4.01–10.4).

Cohen’s ds of about 1 were found for the group comparisons: d = 1.02 for MCI (*n* = 44) versus normal cognition (*n* = 12), and d = 0.97 for moderate (*n* = 14) versus mild dementia (*n* = 37). The ETAM differentiated well between mild cognitive impairment and healthy cognition, with an area under the ROC curve of .83, a sensitivity of .73, a specificity of .83 at the cut-off point of 19, a positive likelihood ratio of 4.29, and a negative likelihood ratio of 0.33. However, as only 10 % of the sample was comprised of unimpaired subjects, this can be seen as only preliminary.

## Discussion

This article describes the validation of a performance test for assessing capacities for performing activities of daily living in persons with MCI and mild dementia. The psychometric parameters determined in 107 study participants showed the measure to be valid and quick to administer and demonstrated its independence from mood.

As mentioned above, there is no validated performance test for determining ADL capacities in mild dementia or MCI to date. Existing performance-based measures that have been validated for moderate to severe dementia are either too easy, too time-consuming, or focus too strongly on cognition. In a review of performance-based measures of functional living skills, Moore et al. suggested that the development of new measures should focus on brevity, should include items determined by patients or caregivers and selected by an empirical procedure, and should be comprised of tasks with “real world functioning” [19]. We tried to follow these recommendations in the development and validation of the ETAM [31].

In contrast to existing measures [30], once translated, the ETAM can in principle be used in all industrialised countries since it does not refer to specific features of the respective healthcare systems. Only the “Traffic situations” item requires adjustment to the road signs typical of the country in question, and the “Finances” item needs to be adjusted to the local currency.

The ICF domain “Communication” was represented on the ETAM only by the “Phone call” item, which had already demonstrated satisfactory psychometric characteristics in the pilot study [31]. Handling phone calls is highly relevant in practice and more future-oriented than, for example, writing a letter. On the ETAM, a mobile phone for older adults that is similar to a cordless telephone is employed. Other performance tests have therefore also included the use of the telephone among their items [27, 30]. It has also been demonstrated in numerous studies that how a person handles the telephone is an important and sensitive indicator of incipient dementia processes. Distinct deficits in this domain of behaviour have been demonstrated in various studies even in individuals with mild cognitive impairment. This also explains the high item difficulty found for this item [7]. In addition to higher organisational skills such as planning to go shopping and paying bills, using the telephone is one of the first areas to show impairment as dementia develops [51]. However, it is to be expected that the item difficulty, which is currently still high, will fall in the next few years as older adults get used to this means of communication. In the present sample, which consisted mainly of participants from the cohorts born between 1930 and 1945, most of the participants were still accustomed to using telephones with circular dials. Particular attention should therefore be paid to this item when the measure is revalidated in the years to come.

The correlation between the ETAM and the B-ADL proved to be weaker than expected. In this context, it is noteworthy that the correlations with the items of a strongly practical nature such as “Washing the dishes” and “Making tea” were particularly low, whereas the correlations with more cognitive items such as “Traffic situations” were stronger. This is likely due to the strong cognitive bias of the B-ADL questionnaire [8]. Reppermund et al. [8] conducted a factor analysis of this measure in a representative sample of 762 older adults. Eleven of the 21 total items loaded on the factor “high cognitive demand”, whereas only nine items were assigned to the factor “low cognitive demand”. In addition, a significant difference between the two study groups “cognitively unimpaired” vs. “MCI” was found only on the factor “high cognitive demand”, whereas the two groups hardly differed at all on the factor “low cognitive demand”. The B-ADL factor therefore probably lacks sensitivity in the upper range of the performance of activities of daily living [8, 52]. A revalidation study should address this and use other measures to determine convergent validity.

The moderate correlation found between the ETAM and the MMSE was consistent with the current international state of the art, according to which the performance of complex instrumental activities of daily living in people with MCI and mild dementia are to a certain extent dependent on cognitive capacity [53]. A minimum level of cognitive capacity is required to be able to carry out complex IADL. Therefore, we did not expect the ETAM test score and the score on a cognition test to be completely independent of one another. This was shown by the coefficient of correlation between the total ETAM score and the total MMSE score (*r* = .46). This moderate association also shows that the ETAM is able to capture a construct that is conceptually distinct and independent of cognition and can be described as instrumental competence in activities of daily living. This also applies to all of the ETAM items that were only moderately correlated with the MMSE, between .19 and .45.

With the current version of the ETAM, we have successfully developed a valid performance test for determining capacities for performing activities of daily living in persons with MCI and mild dementia. The fact that this test is designed specifically for this group of persons is what makes this test unique. The average administration time of 35 min can be considered economical compared with the administration times of other well-known performance tests. The large size of the sample employed must also be emphasised, in comparison with the validation studies of other tests on the performance of tasks of daily living. Due to its orientation towards the ICF, our measure is also based on the current WHO concept of capacities for performing activities of daily living.

However, it should be noted that the discrimination afforded by the ETAM score between persons with MCI and mild dementia and individuals who are completely cognitively healthy on the one hand and between persons with MCI and mild dementia and persons with moderate dementia on the other must be considered preliminary, as the sizes of the sub-samples in our study were small.

Since there is no “gold standard” for determining capacities for performing activities of daily living, the convergent validity had to be tested against an observer-rating scale. The problems associated with observer-rating scales described in the introduction above were also encountered in our study, insofar as the B-ADL was usually completed by the care staff in the supported living accommodations (since either there was no relative available or the relative was also cognitively impaired). The care staff may have found it difficult to assess capacities for performing activities of daily living that were either performed outside of the institution (e.g., the use of public transport) or were generally carried out by the care staff (e.g., the use of domestic appliances, etc.).

Future validation studies should focus on the ETAM’s sensitivity to change and criterion-related validity. An international validation of versions in other languages is also desirable.

## Conclusions

The ETAM test proved to be a valid, reliable, and feasible performance-based assessment for ADL capabilities in persons with mild dementia or MCI. It is therefore suitable for use in both clinical practice and research.

### Consent for publication

Written informed consent for publication of their images was obtained from the participant. A copy of the consent form is available for review by the Editor of this journal.

### Availability of data and materials

All data are contained within the manuscript and its additional files.

## Additional files

Additional file 1: Material for carrying out the ETAM. (DOCX 4613 kb) Additional file 2: ETAM evaluation and documentation form. (DOC 82 kb)

## Abbreviations

ADCS-MCI-ADL: Alzheimer’s Disease Cooperative Study Scale for ADL in Mild Cognitive Impairment
ADL: activities of daily living
B-ADL: Bayer Activities of Daily Living Scale
DAFA: direct assessment of functional abilities
DAFS: direct assessment of functional status
ETAM: Erlangen Test of Activities of Daily Living in Persons with Mild Dementia and Mild Cognitive Impairment
FLSA: functional living skills assessment
GDS-15: geriatric depression scale 15
IADL: instrumental activities of daily living
ICF: International Classification of Functioning and Health
MCI: mild cognitive impairment
MMSE: mini mental state examination
MoCA: Montreal cognitive assessment
SAILS: structured assessment of independent living skills
TEFA: test of everyday functional abilities
