# Supplementary material for: Validation of the Erlangen Test of Activities of Daily Living in Persons with Mild Dementia or Mild Cognitive Impairment (ETAM)
Source: BMC Geriatr. 2016 May 26;16:111. doi: 10.1186/s12877-016-0271-9 (PMC4882865; doi:10.1186/s12877-016-0271-9)
Supplement: Additional file 1: — Material for carrying out the ETAM. (DOCX 4613 kb) [file 12877_2016_271_MOESM1_ESM.docx]

**Material for carrying out the ETAM**

| Task 1: Medication |  |
| --- | --- |
| 1 Medicine dispenser for one day with four compartments labelled  “morning, midday, evening, night”  1 Pack Strepsils (over the counter product)  1 Pack Magnesium tablets (over the counter product)  1 Pack Ibuprofen (over the counter product): Content replaced by placebos (over the counter products)  Remark: The tablets can be replaced with others which are freely available and can be easily distinguished. In this case, please make sure that NO HARM can be caused to the participant if they accidentally swallow them. Dietary supplements or placebos should be the first choice. In this case the instructions have to be adjusted. | not included  not included  not included  not included |
| Task 2: Make tea |  |
| 1 kettle: maximum capacity around 0.5l with extra on switch, if the interview takes place at the home of the participant use the kettle there and skip the instruction.  1 cup: maximum capacity 0.2l  2 varieties of tea  1 small bowl for used tea bags | not included  not included  not included  not included |
| Task 3: Traffic |  |
| Six photos of everyday traffic situations with a question underneath regarding the right of way in traffic (see next pages, only suitable for right-hand side traffic!) | Pages 5-11 of this document |
| Task 4: Alarm clock |  |
| 1 Standard alarm clock with a knob on the back which when extended can be used to change the time; marking of the knob with a special symbol (see “Instructions for tasks” on the next pages) | not included |

For each item, you will also need a print-out of the task instructions in big fonts which you place in front of the participant so it is easily visible. You will find them on the following pages.

| Task 5: Finances |  |
| --- | --- |
| 3 self-made fictional advertising leaflets showing a maximum of two products each; each leaflet must advertise a pack of butter (see following pages)  1 standard pen  1 sheet of blank paper  coins amounting to EUR 2.28 (adjust to your currency), divided as follows: | Pages 14-16  not included  not included  not included |
| \| **Contents** \| Right solution to the test: \| \| --- \| --- \| \| **1x 1 Euro** \| 1x 1 Euro \| \| **1x 50 Cent** \| 1x 50 Cent \| \| **3x 20 Cent** \| 2x 20 Cent \| \| **1x 10 Cent** \| 1x 10 Cent \| \| **3x 2 Cent** \| 2x 2 Cent \| \| **2x 1 Cent** \| 2x 1 Cent \| \|  \| Total: EUR 2.05 \| |  |
| Task 6: Telephoning |  |
| 1 standard mobile for senior citizens with a red end-call and a green accept-call button together with a SIM card with sufficient credit  1 standard pen  1 sheet of blank paper  1 sheet with telephone numbers (see next pages)  1 voicemail announcement of an actually existing number which is listed in the telephone list (text of the announcement: *You are connected with the practice of Dr. Miller. Unfortunately, you are calling outside our consultation hours which are between 8 and 10 in the morning and 2 and 6 on Tuesday afternoon. Please try to contact us during our consultation hours.* | not included  not included  Pages 18-19, please insert number of the voicemail announcement! |

| 1. **Task medicine** |
| --- |

On the table in front of you, you can see a pill box and three different packs of medicine.

Sort the pills into the pill box:

- Strepsils: one every morning and evening
- Magnesium: one every morning, midday and night
- Ibuprofen: one every the morning

| 1. **Task tea** |
| --- |

On the table in front of you, you can see a cup, a kettle and different kinds of tea.

Please make yourself a cup of tea.

Feel free to choose the kind of tea you like. Only put water for one cup into the kettle.

Ask me for help if you have problems carrying the full kettle.

| 1. **Task traffic** |
| --- |

I will now show you six photos of everyday traffic situations.

Take your time to look at them and answer each of the questions below them.

Tell me your decision and your reasons for it.


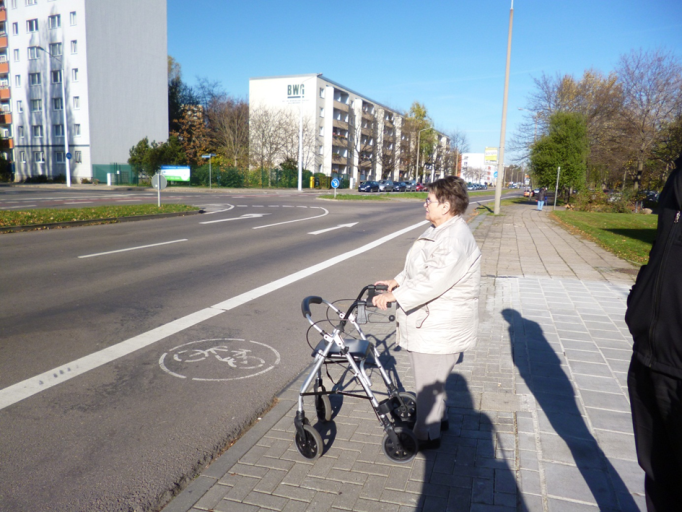


What does the woman on the photo have to consider before she can cross the street without problems?

1. Cars
2. Cars and bicycles


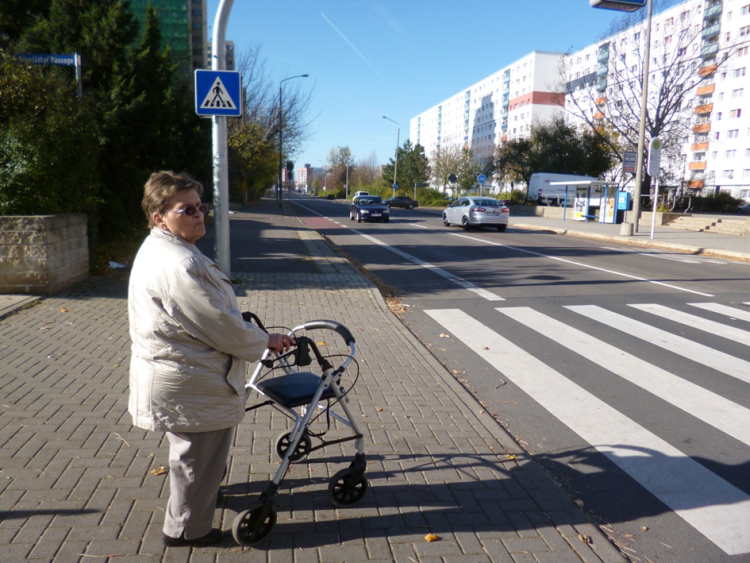


Who has right of way in this situation - the approaching car or the woman?


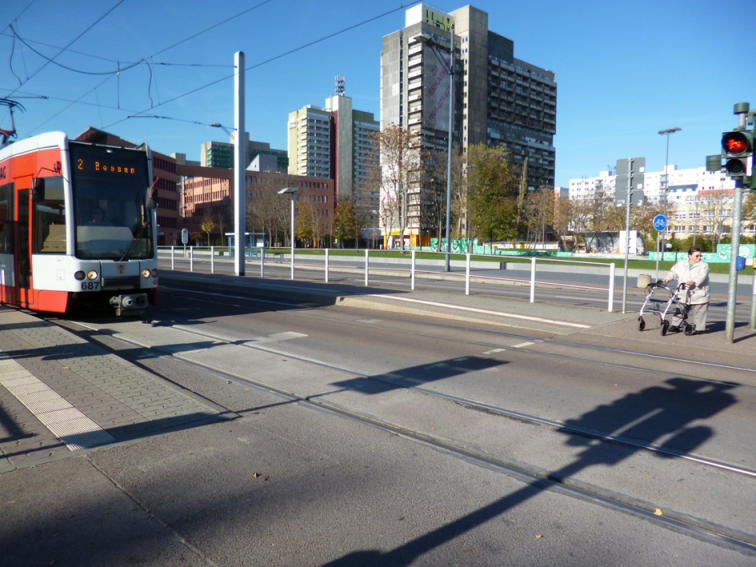


Who has right of way in this situation? The woman or the tram?


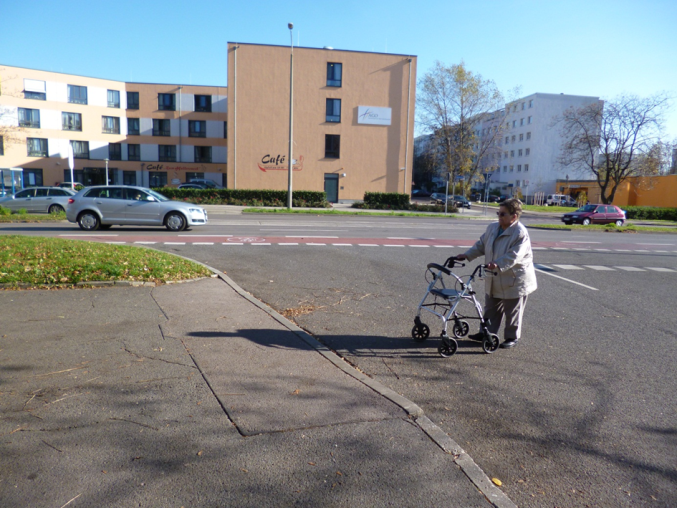


The car wants to turn right into the road. Who has right of way?

1. Car
2. Woman


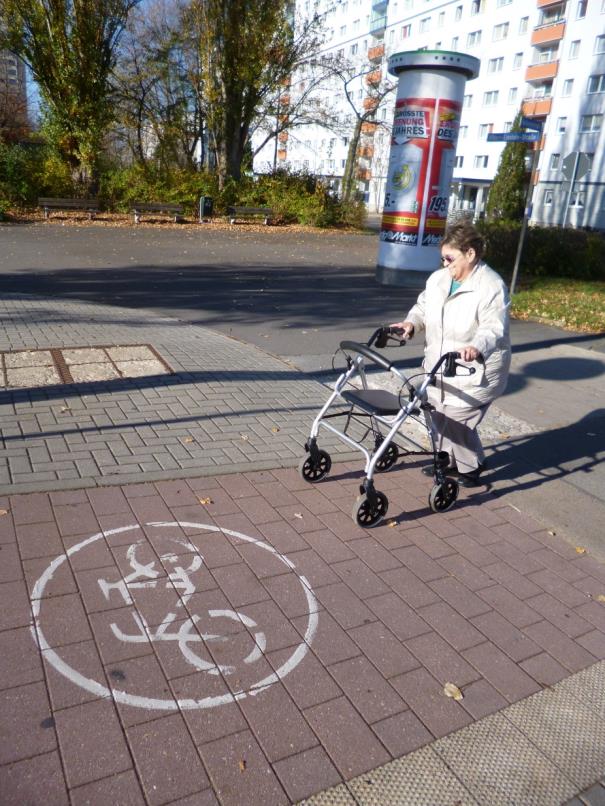


Is what the woman on the photo is doing right or wrong?


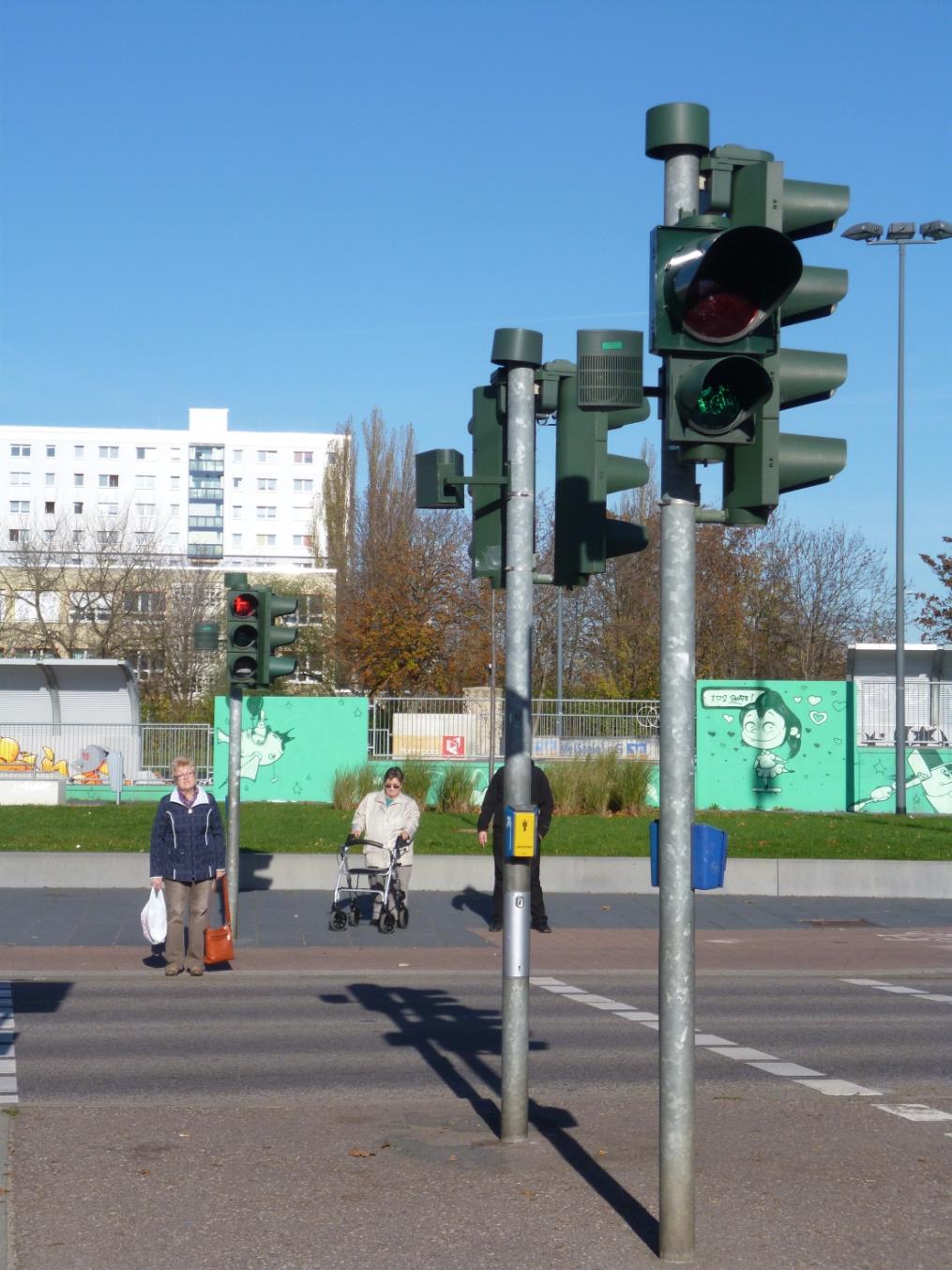


Are the two women allowed to cross the street immediately or do they have to wait?

| 1. **Task alarm clock** |
| --- |

Please read the time that is now set on the clock.

Please change the clock to 1:45 pm. To do this, pull out the knob with the symbol.

| 1. **Task finances** |
| --- |

On the table in front of you there are three advertising leaflets, pen and paper and a stack of coins.

You want to buy three grocery items:

- one pack of butter
- one litre of milk
- one bread roll

Pick the cheapest offer for butter from the three leaflets.

Calculate how much money you need for all the products together.

Take the necessary amount from the stack of coins in front of you.

Alli

cheap and good


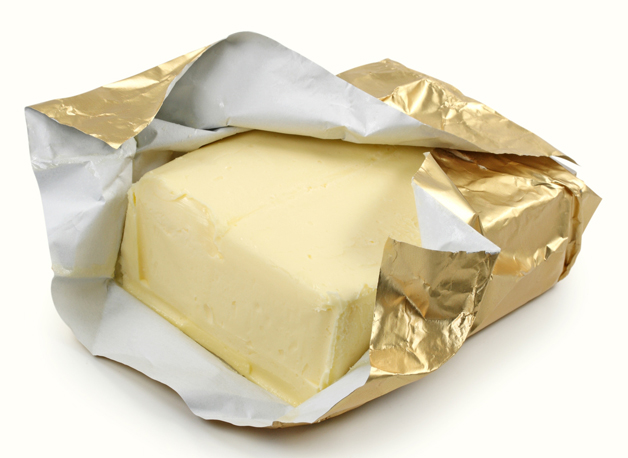


**€ 1.20**


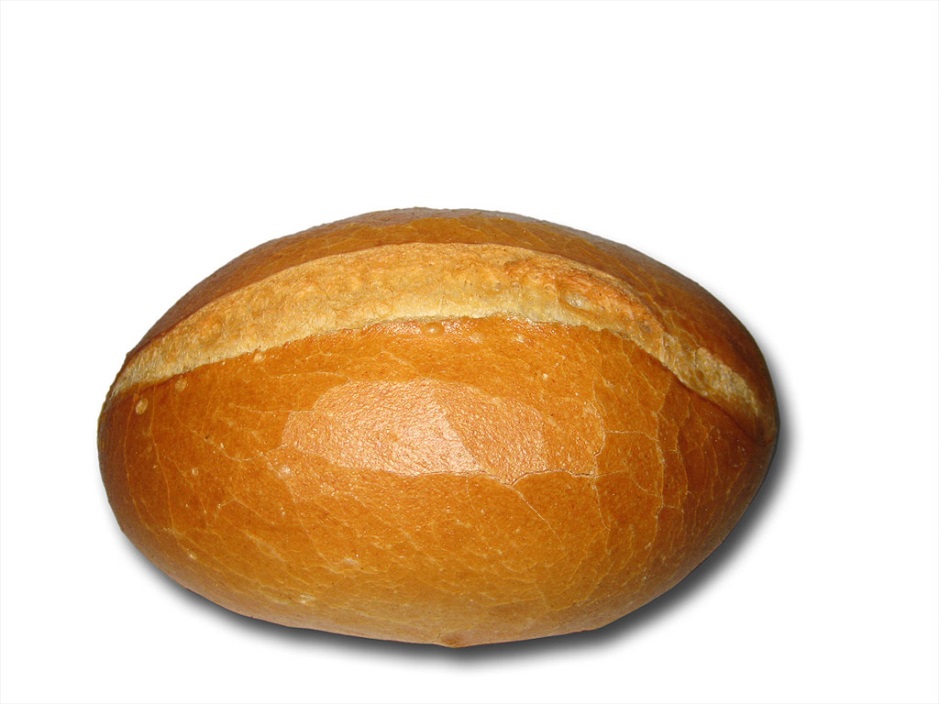


**€ 0.25**

Your Grocery Store


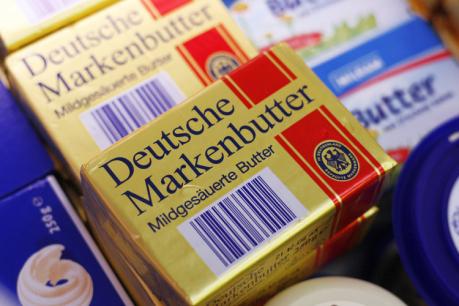


**only 1 Euro**


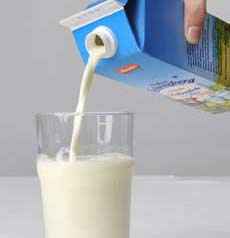


**only 80 Cent**

Buy Clever


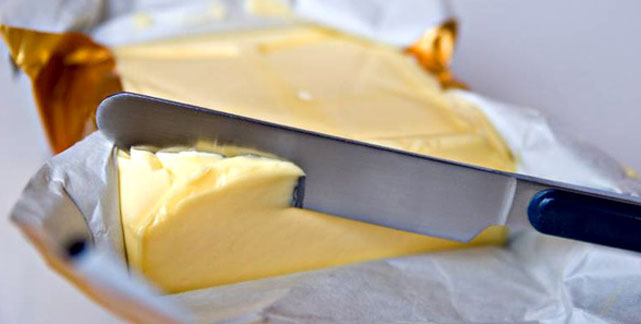


**for an unbeatable €2**

**for an unbeatable €2**

**for an unbeatable €2**

**for an unbeatable €2**

**for an unbeatable €2**

| 1. **Task telephoning** |
| --- |

On the table in front of you there is a telephone, pen and paper and a sheet with telephone numbers.

Please find the number of Dr. Miller in the phone list.

Dial the number and get the information about his consultations hours on Tuesdays from the voicemail.

Note the consultation hours on a piece of paper.


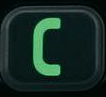

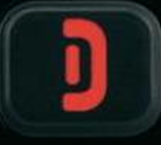


Telephone list

| Anthony, Charles 09783/9286463 |
| --- |
| Arnold, Francis 09172/5792415 |
| Bobbin, Maggie 9468/93982472 |
| Bond, Stephen 90324/3029572 |
| Butterfield, Anna 04395/2590913 |
| Camper, Justin 23140/0193175 |
| Cleese, Jonathan 13456/0935892 |
| Cunningham, Andrew 48540/0239503 |
| Dalton, Paul 934844/9488998 |
| Dexter, Caroline 947934/0212344 |
| Domasius, Natalie 23974/33123004 |
| Finley, Amanda 34468/03495069 |
| Finnigan, Stewart 5445/054244666 |
| Freeman, Robert 55598/23421578 |
| Giles, Arnold 32535/l3233244 |
| Gillingham, Eve 4034/214943445 |
| Hunter, Richard 54545/22404657 |
| Idle, Benedict 43250/23495890 |
| Jones, Catherine 3557/325957985 |
| Jordan, Roberta 45577/05059312 |
| Lovich, Sophie 59004/3450455 |
| Letterman, Eric 35869/09495830 |
| Miller Dr., Robert The number of your voicemail! |
| Newman, Eileen 456436/0359035 |
| O’Connor, Louis 325657/3245050 |
| Peters, David 453678/30957902 |
| Roberts, Sarah 49590/94589823 |
