# Supplementary material for: Validation of the Erlangen Test of Activities of Daily Living in Persons with Mild Dementia or Mild Cognitive Impairment (ETAM)
Source: BMC Geriatr. 2016 May 26;16:111. doi: 10.1186/s12877-016-0271-9 (PMC4882865; doi:10.1186/s12877-016-0271-9)
Supplement: Additional file 2: — ETAM evaluation and documentation form. (DOC 82 kb) [file 12877_2016_271_MOESM2_ESM.doc]

**ETAM evaluation and documentation form**

**General evaluation information**

1. A print-out of the instructions is presented to the participant to help him/her remember. Please make sure the font size is big enough (see material).
2. The participants may be told everything that is on the instruction sheets several times, however, they must not be given any additional information and no gestures must be used (for example pointing at clues).
3. Once the participant replies clearly, that reply is counted, be it right or wrong. Please do not say anything like “come on, think that through again” after that.
4. If the participant, after getting asked three times does not try to act, the next task is started and the abandoned one rated with 0 points. Check “refused”, if applicable.
5. The following applies for the tasks ‘finances’ and ‘telephoning’: The letters and figures don’t have to be written perfectly. However, it has to be possible to decipher what was written down or the participant has to give the answer orally in a correct way.
6. In case of impairments which prevent the execution of an item such as not being able to open the medication package or to operate the kettle the item is rated with 0 points. Nevertheless, you can help the participant if he/she asks you so, but do NOT rate this (0 points).
7. For all linked subsequent items the following applies:

If the necessary intermediate step, for example typing in the telephone number cannot be mastered, the task will be abandoned. Only the points acquired until then will be noted.

Examples:

- - 1. Pill organizer: participant is not able to open the box
    2. Tea: participant is not able to fill water in the kettle or turn it on
    3. Alarm clock: participant is not able to put the knob out or turn it

If the participant fails in a step that is not necessary for proceeding with the task, then the participant can continue completing the task

Examples:

1. Finances: if he/she miscalculates the necessary amount of money, he/she can take this amount from the stack;
2. Alarm clock: if the time is not given correctly, he/she can still set the correct time in the next step.

**ETAM evaluation and documentation form**

Surname: ______________________, First name: _____________________

Location: __________________, Date: _______________________

Start of the test: _______ End of the test: ________

Examiner: _______________________

**Prerequisites for the feasibility of the test**

 impaired vision  has reading glasses

 impaired hearing  has hearing aid

 weakness or impaired movement of the ‘writing hand’

**Test results**

**I. Pill organizer**

Material: three packs of medicine (Strepsils, Magnesium and Ibuprofen), pill box

Instruction: Sort the pills into the pill box:

- Strepsils: one every morning and evening
- Magnesium: one every morning, midday and night
- Ibuprofen: one every morning

Times instruction was given:  1 x  2 x  3 x

Refused to execute task: 

(not if not able to execute task)

Evaluation rules:

- For each correctly executed subtask:1 point,
- If participants sorts the pills only randomly: 0 points
- For every pill sorted wrongly: drawback 1 point

a) Strepsils morning 

b) Strepsils evening 

c) Magnesium morning 

d) Magnesium midday 

d) Magnesium night 

f) Ibuprofen morning 

**Points Sum I: ______ (max. 6 points)**

**II. Tea**

Material: Institute’s or personal kettle, tea bags, one cup, one little bowl for used tea bags, if necessary an extension cord.

Instruction: On the table in front of you, you can see a cup, a kettle and different kinds of tea. The on switch for the kettle is here (point to it). Please make yourself a cup of tea. Feel free to choose the kind of tea you like. Ask me for help if you have problems carying the full kettle.

*Note: If the participant is not familiar with the kettle, show them where the on switch is. Only let the participant pour water for one cup of tea into the kettle. If necessary, stop the participant from putting in more (no consequences on the score). Wait until the water boils. Make sure the participant does not get into direct contact with the steam and hot water.*

Times instruction was given:  1 x  2 x  3 x

Refused to execute task: 

(not if not able to execute task)

Evaluation (1 point for each correctly executed subtask):

a) Put in water and switch on kettle 

b) Put tea bag into the cup 

c) Add water 

**Points sum II: ______ (max. 3 points)**

**III. Traffic situation**

Material: Traffic situations

Instruction: I will now show you six photos of everyday traffic situations. Take your time to look at them and answer each of the questions below them. Tell me your decision and your reasons for it.

Times instruction was given:  1 x  2 x  3 x

Refused to execute task: 

(not if not able to execute task)

Evaluation (1 point for each correctly executed subtask):

*Note: Scoring only when the reason was right, please note the reason.*

a) Traffic situation1-reason: b; because of the bicycle path 

b) Traffic situation2-reason: woman; because of the zebra crossing 

c) Traffic situation3-reason: tramway; because of the red traffic light for pedestrians 

d) Traffic situation4-reason: woman; the one turning has to wait the woman is already crossing 

e) Traffic situation5-reason: wrong; she is walking on the bicycle path 

f) Traffic situation6-reason: wait; there is a red traffic light 

**Points sum III: ______ (max. 6 points)**

**IV. Alarm clock**

Material: Alarm clock

ATTENTION: Before the task is started, make sure, the clock is set to 10:30 am and the turn knob is pushed in (i.e. it is flush).

Instruction: Please read the time that is now set on the clock.

Please change the clock to 1:45 pm. To do this, pull out the knob with the symbol (point at the symbol on the paper).

Times instruction was given:  1 x  2 x  3 x

Refused to execute task: 

(not if not able to execute task)

Evaluation (1 point for each correctly executed subtask):

a) Time read (10:30 am) 

b) Knob pulled out 

c) Time set correctly (15 min deviation allowed) 

**Points sum IV: ______ (max. 3 points)**

**Handling finances**

Material: Three advertising leaflets, pen, paper, envelop with coins

Instruction: On the table in front of you you can see three advertising leaflets, pen and paper and a stack of coins. You want to buy three grocery items: One pack of butter, one litre of milk and one bread roll. Pick the cheapest offer for butter from the three leaflets.

Calculate how much money you need for all products together. Take the necessary amount from the stack of coins in front of you.

Times instruction was given:  1 x  2 x  3 x

Refused to execute task: 

(not if not able to execute task)

Evaluation (1 point for each correctly executed subtask):

a) Recognised the butter in all three leaflets (ask participant to indicate them) 

b) Cheapest offer for butter found 

c) Correct amount for milk recognised 

d) Correct amount for the roll recognised 

e) Added-up correctly (right answer: EUR 2.05) (or equivalent amount in your currency) 

f) Correct amount taken from stack of coins (if e) is wrong, give correct amount) 

**Points sum V: ______ (max. 6 points)**

**VI. Telephoning**

Material: Pen, paper, sheet with telephone numbers, mobile phone for seniors

Instruction: On the table in front of you there is a telephone, pen and paper and a sheet with telephone numbers.

You can key in a telephone number of your choice with the keypad (point at it). The numbers you keyed in appear on the display (point at it). After you have checked the number, you can start the call by pressing the green key (point at it). When you want to end the call press the red button (point at it). Let the participant practice once with the number combination ‘333’ without letting the phone ring

Instruction: Please find the number of Dr. Miller in the phone book. Dial the number and get the information about his consultations hours on Tuesdays from the voicemail. Note the consultation hours on a piece of paper.

Times instruction was given:  1 x  2 x  3 x

Refused to execute task: 

(not if not able to execute task)

Evaluation (1 point for each correctly executed subtask):

*Note: If the senior citizen is not able to dial the number, abandon the task:*

*Points for f) (reproduction of the consultation hours) only if time in the morning (8-10 am) and evening (2-6 pm) are correctly named.*

a) Found correct telephone number 

b) Keyed in number 

c) Pressed green button 

d) Listened completely 

e) Ended phone call 

f) Reproduced consultation hours 

**Points sum VI: ______**

**(max. 6 points)**

**Total score: ______**

**(max. 30 points)**
